# Foam Cell Formation *In Vivo* Converts Macrophages to a Pro-Fibrotic Phenotype

**DOI:** 10.1371/journal.pone.0128163

**Published:** 2015-07-21

**Authors:** Anita C. Thomas, Wouter J. Eijgelaar, Mat J. A. P. Daemen, Andrew C. Newby

**Affiliations:** 1 Bristol Heart Institute, University of Bristol, Bristol, United Kingdom; 2 Cardiovascular Research Institute Maastricht (CARIM), Maastricht, The Netherlands; 3 Academisch Medisch Centrum (AMC), Amsterdam, The Netherlands; Medical Faculty, Ludwig Maximilians University Munich, GERMANY

## Abstract

Formation of foam cell macrophages, which sequester extracellular modified lipids, is a key event in atherosclerosis. How lipid loading affects macrophage phenotype is controversial, with evidence suggesting either pro- or anti-inflammatory consequences. To investigate this further, we compared the transcriptomes of foamy and non-foamy macrophages that accumulate in the subcutaneous granulomas of fed-fat ApoE null mice and normal chow fed wild-type mice in vivo. Consistent with previous studies, LXR/RXR pathway genes were significantly over-represented among the genes up-regulated in foam cell macrophages. Unexpectedly, the hepatic fibrosis pathway, associated with platelet derived growth factor and transforming growth factor-β action, was also over-represented. Several collagen polypeptides and proteoglycan core proteins as well as connective tissue growth factor and fibrosis-related FOS and JUN transcription factors were up-regulated in foam cell macrophages. Increased expression of several of these genes was confirmed at the protein level in foam cell macrophages from subcutaneous granulomas and in atherosclerotic plaques. Moreover, phosphorylation and nuclear translocation of SMAD2, which is downstream of several transforming growth factor-β family members, was also detected in foam cell macrophages. We conclude that foam cell formation in vivo leads to a pro-fibrotic macrophage phenotype, which could contribute to plaque stability, especially in early lesions that have few vascular smooth muscle cells.

## Introduction

Atherosclerosis, the underlying cause of many vascular diseases, can be considered to be a healing response to multiple injurious stimuli that include endothelial dysfunction, lipid retention and inflammation (with activation of both innate and adaptive immune systems) [1,2]. Retention and oxidation of extracellular lipid in the vessel wall provokes the production of inflammatory mediators that recruit monocytes, which differentiate to macrophages [3,4]: it also creates neo-epitopes that can activate the immune system. Modified extracellular lipid is then taken up into foam cell macrophages (FCMs) [3] and smooth muscle cells (SMCs) [5,6]. Plaque macrophages and FCMs may also be differentially activated, adopting pro-inflammatory phenotypes similar to those designated M1 or ‘classically activated’ from *in vitro* experiments [7–9]. M1-like macrophages have been observed especially at the shoulder region of the fibrous cap of plaques. They express higher levels of many proteases, including matrix metalloproteinases (MMPs) [8], which can degrade the extracellular matrix leading to loss of collagen. This promotes subsequent cap rupture, thrombus formation and hence life-threatening myocardial infarctions or strokes [10,11]. Death of FCMs, because it releases lipid and enlarges the necrotic core of the plaque, increases mechanical stress on the plaque cap [12] and further promotes plaque rupture. Conversely, plaque macrophages with similarities to M2 or alternatively activated phenotypes have also been detected, especially in areas of plaques remote from the core, and in the adventitia [3,9,13]. These include so-called M2a, M2c/Mreg, Mhem and Mox macrophages that are anti-inflammatory, promote tissue repair and hence favour plaque stability [3,4,14–16].

The role of lipid uptake and foam cell formation *per se* in the activation of macrophage behaviour that provokes plaque rupture is controversial. Different *in vitro* studies suggested pro- or anti-inflammatory effects of treatment with oxidised lipids, whereas others showed no major influence on markers of M1 and M2 polarization [17–20]. Results from *ex vivo* studies of FCMs generated *in vivo* are also conflicting. Rabbit FCMs purified from subcutaneous granulomas showed increased activation of the nuclear factor-κB (NF-κB) pathway and production of several NF-κB dependent MMPs compared with non-foamy macrophages (NFMs) [21,22]. On the other hand, a recent paper that compared FCMs with NFMs isolated from the peritoneum of atherosclerosis-prone mice suggested that increased desmosterol levels in FCMs initiate a transcriptional programme mediated by the liver X receptor (LXR) that suppresses the expression of many pro-inflammatory genes [23]. To re-examine this controversy, we adapted the subcutaneous sponge model we used in rabbits [21,22] for use in mice, and compared the transcriptomes of FCMs and NFMs produced in fat-fed ApoE null or wild-type mice. We then validated the differential expression of selected genes at the protein level both in subcutaneous granulomas and in brachiocephalic artery plaques.

We found, unexpectedly, that foam cell formation under these conditions favoured neither M1 nor M2 polarization but instead induced a pro-fibrotic transcriptional programme that should favour plaque stability. Understanding how FCMs are different from NFMs could enhance our ability to treat or prevent atherosclerotic plaque development and subsequent acute events.

## Materials and Methods

### Animals

The housing and care of all animals and procedures used in these studies was in accordance with and under license of the Animals (Scientific Procedures) Act 1986 (London, United Kingdom), and conform to the Guide for the Care and Use of Laboratory Animals published by the U.S. National Institutes of Health (Publication No. 85–23, revised 1996). The study received local institutional review board (University of Bristol, Bristol, United Kingdom) approval. Homozygous ApoE^−/−^ mice and wild-type (control) mice on a C57BL background were bred in the University of Bristol Animal Services Unit.

#### Surgical sponge implantation and harvest of foamy and non-foamy macrophages

Adult male ApoE^−/−^ mice (10–25 weeks old) were changed from normal rodent diet to a high fat diet containing 23% fat from lard, supplemented with 0.15% (w/w) cholesterol (Special Diets Services, UK). Three weeks after commencing the high-cholesterol diet, the animals were anaesthetised with halothane and had six 0.5 cm^3^ pieces of sterile sponges (containing Matrigel (BD Biosciences, UK)) placed dorsally, subcutaneously under aseptic conditions and buprenorphine analgesia. The high-fat diet continued for 4 weeks post-surgery, when the sponges were recovered. For controls, age-matched male C57/129 mice were fed a normal chow diet before and after sponge insertion as above. Water and food were given *ad libitum*. Mice were euthanized by an overdose of halothane and the sponges removed using aseptic technique.

FCMs were purified from sponges removed from fat-fed ApoE null mice based on their lower buoyant density than other cells followed by differential adherence to plastic, whereas NFM were purified by differential adherence alone [21,22]. Purified cells were used for RNA extraction (Illumina array, RT-qPCR), viability (Trypan blue), lipid content (histochemistry) or identification of cell type (RT-qPCR, immunocytochemistry). Some whole sponges from ApoE^-/-^ mice were fixed in 10% phosphate-buffered formalin and paraffin-embedded; 3 μm sections were then cut and used for immunohistochemistry.

#### Development of atherosclerosis

ApoE^-/-^ male mice commenced a high-fat diet at 5 weeks of age. Twelve weeks later the animals were killed and perfusion fixed at normal pressure with phosphate-buffered saline (PBS) followed by 10% phosphate-buffered formalin. Brachiocephalic arteries were dissected out as described previously [24] and embedded in paraffin. Sections (3 μm thick) were prepared and used for immunohistochemistry.

### Transcriptomic comparison of FCMs and NFMs

Total cellular RNA was extracted using the Qiagen RNease MiniKit (UK) according to the manufacturer’s instructions. RNA concentration was determined using NanoDrop spectrometry (NanoDrop Technologies, USA). RNA samples of high quality (A260/280>2) (n = 4) were compared using Illumina bead chips (MouseRef8 v2.0 Expression BeadChips, Illumina, USA) at 1 μg/sample.

#### Functional Annotation

All genes from the dataset that met the unadjusted P-value cut-off of 0.01 were uploaded to the Ingenuity Pathways Analysis (Ingenuity Systems, www.ingenuity.com) system and included in the analysis. Each identifier was mapped to its corresponding object in the Ingenuity Knowledge Base. Functional analysis identified the biological functions and canonical pathway analysis identified the pathways that were most significant to the data set. Network maps were also generated within Ingenuity Pathway Analysis. Differentially expressed genes were overlaid onto a global molecular network developed from information contained in the Ingenuity Knowledge Base and networks of these molecules algorithmically generated based on their connectivity. Molecules are represented as nodes, and the biological relationship between two nodes is represented as an edge (line). The intensity of the node colour indicates the degree of up- (red) or down- (green) regulation. Nodes are displayed using various shapes that represent the functional class of the gene product. Edges are displayed with various labels that describe the nature of the relationship between the nodes (e.g., P for phosphorylation, T for transcription).

Differentially expressed genes were also submitted to GO annotation and clustering using DAVID Bioinformatics Resources (National Institute of Allergy and Infectious Diseases (NIAID) 2008, NIH, http://david.abcc.ncifcrf.gov).

#### RNA isolation and quantitative reverse transcriptase polymerase chain reaction (RT-qPCR) assays

The array results were validated and expanded on RNA samples prepared in the same way using RT-qPCR (n = 5–7) with selected primer pairs (Table 1). For reverse transcription, 100 ng of total RNA was used to make cDNA, using a Qiagen Quantitect Reverse Transcripase Kit according to the manufacturer’s instructions. Sequences of the PCR primer pairs used to amplify the respective cDNAs were designed using Ensembl and Primer3, and the specificity of the sequence confirmed using Nucleotide blast (NCBI). Samples were compared, either after normalising to a housekeeping gene (36B4) or as copies per ng RNA.

**Table 1:** Primer sequences used in this study.

GENE	ALTERNATIVE NAME	SPECIES	PRIMER SEQUENCE
36B4	ribosomal protein, large, P0, RPLP0	human	GCCCAGGGAAGACAGGGCGA GCGCATCATGGTGTTCTTGCCCA
NR2B2	retinoid X receptor β, RXRβ	mouse	GGTGCTGACAGAGCTAGTGTCCAA TGCTTGCAATAGGTCTCCAGTGAG
NR2B1	RXRα	mouse	AGGACAGTACGCAAAGACCTGACC ATGTTTGCCTCCACGTATGTCTCA
NR1H3	liver X receptor α, LXRα	mouse	GCAGGACCAGCTCCAAGTAGAGAG CACAAAGGACACGGTGAAACAGTC
NR1H2	LXRβ	mouse	GGCGGCCCAACTGCAGTGCAACAA GCAAAGCGTTGCTGGCGGGCATCT
SREBF1	sterol regulatory element-binding transcription factor-1, SREBP1	mouse	AGGCCATCGACTACATCCG TCCATAGACACATCTGTGCCTC
FASN	fatty acid synthase	mouse	GGCTCTATGGATTACCCAAGC CCAGTGTTCGTTCCTCGGA
MSR1	macrophage scavenger receptor-1, SR-A1, SCARA1	mouse	GCTGCCCTCATTGCTCTCT CTGGAAGCGTTCCGTGTCT
CD36	thrombospondin receptor	mouse	GTACAGCCCAATGGAGCCA AACCCCACAAGAGTTCTTTCAAA
SCARB1	scavenger receptor B1, SR-B1	mouse	GGTGCGCCTCTGTTTCTCTC AGAACTACTGGCTCGATCTTCCCT
ABCA1	ATP-binding cassette, sub-family A1	mouse	CTCAGTTAAGGCTGCTGCTG TCAGGCGTACAGAGATCAGG
PPARγ	peroxisome proliferator-activated receptor-γ	mouse	TTGACAGGAAGGACAACGGACAAA TGTGATCTCTTGCACGGCTTCTAC
COL1α1	collagen 1α1	mouse	GATGATGCTAACGTGGTTCGTGAC CCATGTTGCAGTAGACCTTGATGG
COL4α1	collagen 4α1	mouse	CTGGCACAAAAGGGACGAG CGTGGCCGAGAATTTCACC
COL6α1	collagen 6α1	mouse	ATGTGACCCAACTGGTCAACTCAA AGCATGGAAGACAGAACACAGACG
COL8α1	collagen 8α1	mouse	TCATCATTTCCCTGAACTCTGTC CAAAGGCATGTGAGGGACTTG
BGN	biglycan	mouse	TAGCCAGTCCCTCCACAAACAAAT AGGAAGCTCCTTGATCCTCGTCTT
DCN	decorin	mouse	CACAACCTTGCTAGACCTGC GAAGTTCCTGGAGAGTTCTGG
CTGF	connective tissue growth factor	human	GGTGTACCGCAGCGGAGAGT GGGCCAAACGTGTCTTCCAG
BMP1	bone morphogenic protein v1,2	mouse	AGACCTTTATTCCCATGCCCAGTT TTCTTGGAGATGGTGTCGTCAGAG
THBS1	thrombospondin-1	mouse	GGGCAAAGAACTTGTCCAGACTGT ACTGGGTGACTTGTTTCCACATCA
TGFβ1	transforming growth factor-β1	mouse	CTCCCGTGGCTTCTAGTGC GCCTTAGTTTGGACAGGATCTG
FOS	FBJ murine osteosarcoma viral oncogene homolog	mouse	TTCGACCATGATGTTCTCG TTGGCACTAGAGACGGACAGA
FosB	FosB	mouse	CCCTCCTGCATATCTTTGTCCTGT CTGTCATTTCCTCGTTGGGTCTCT
JUN	jun proto-oncogene	mouse	TGACTGCAAAGATGGAAACGA CAGGTTCAAGGTCATGCTCTGT
JunB	JunB	mouse	TCACGACGACTCTTACGCAG CCTTGAGACCCCGATAGGGA
SMAD2	Smad2 v1,2	mouse	ATGTCGTCCATCTTGCCATTC AACCGTCCTGTTTTCTTTAGCTT
SMAD3	Smad3	mouse	TGCACAGCCACCATGAATTAC TCCATCTTCACTCAGGTAGCC
ACVR1	activin A receptor-1/ALK2 v1,2,3	mouse	AATGGTGAGCAATGGTATAGTG GGGTCTGAGAACCATCTGTTAGG
TBFBR1	TGFβ receptor-1/ALK5	mouse	CAGCTCCTCATCGTGTTGGTG GCACATACAAATGGCCTGTCTC
CTSC	cathepsin C	mouse	GCAGGTCATCTACAATGCAACCAG TGGAGCATAAATGCTTCTAAGGGA
CTSE	cathepsin E	mouse	GCAGGTCATCTACAATGCAACCAG TGGAGCATAAATGCTTCTAAGGGA
CTSB	cystatin b	mouse	AGGTGAAGTCCCAGCTTGAAT TCTGATAGGAAGACAGGGTCA
CSTF	cystatin f	mouse	TGTTCCAAAGATTTGATCTCCAG GTACCAGGGCTTTGCTGACAT
LYZ	lysozyme v1&2	mouse	GAATGCCTGTGGGATCAATTGC GCTGCAGTAGAAGCACACCG
MMP2	matrix metalloproteinase-2	mouse	GGCTGACATCATGATCAACTTTGG GCCATCAGCCGTTCCCATACTTTAC
MMP9	MMP9	mouse	AGAGAGGAGTCTGGGGTCTGGTTT GAGAACACCACCGAGCTATCCACT
MMP12	MMP12	mouse	AATTACACTCCGGACATGAAGCGT GGCTAGTGTACCACCTTTGCCATC
MMP13	MMP13	mouse	ATGATGATGAAACCTGGACAAGCA ATAGGGCTGGGTCACACTTCTCTG
MMP14	MMP14	mouse	ACCACAAGGACTTTGCCTCTGAAG CACCGAGCTGTGAGATTCCCTTGA
MMP23	MMP23	mouse	CAAGGTTGGTGAGAGAGGGTAGGA AGGAGTAGGTGCTGAGAACACGCT
TIMP1	tissue inhibitor of metalloproteinase-1	mouse	AGGAACGAAATTTGCACATCAGT CAAAGTGACGGCTCTGGTAGTCCT
TIMP2	TIMP2	mouse	GACTCCCCCTCAGACTCTCCCTAC CATATTGATACCACCGCACAGGAA
TIMP3	TIMP3	mouse	CACATCAAGGTGCCATTCAGGTAG GTTCTCTCCTCCTCAACCCAAACA
NOS2	nitric oxide synthase-2, iNOS	mouse	CTCATGACATCGACCAGAAGCGT TATATTGCTGTGGCTCCCATGTTG
IL-6	interleukin-6	mouse	GTTCTCTGGGAAATCGTGGA TTCTGCAAGTGCATCATCGT
CCL2	chemokine (C-C motif) ligand-2, MCP1	rabbit	GCTTGCCCAGCCAGATGCCGTGAA GGTTGGCAATGGCATCCTGGACCC
NF-κB1	nuclear factor of kappa light chain polypeptide gene enhancer in B-cells 1	mouse	GGAGGCATGTTCGGTAGTGG CCCTGCGTTGGATTTCGTG
ARG1	arginase-1	mouse	AGTCTGGCAGTTGGAAGCATCTCT TTCCTTCAGGAGAAAGGACACAGG
ARG2	arginase-2	mouse	ACAGCCAGACTAGCACTGGATGTC CGAATGCCTTGCAACTCTGTAATG
Chi3l3	chitinase-like 3, Ym1	mouse	CAGGTCTGGCAATTCTTCTG GTCTTGCTCATGTGTGTAAGTG
CD206	mannose receptor, MRC1	mouse	CCATTTATCATTCCCTCAGCAAGC AAATGTCACTGGGGTTCCATCACT
RND3	member Rho GTPase family	human	GGGACACTTCGGGTTCTCCTTACT TGGACAAAATTCCTGGATTTCACC
SARAF	store-operated calcium entry-associated regulatory factor, Tmem66	mouse	GGCTTTAAGTCGGAGTTCACAGGA TCGAGTCTGCATTAGAGGATGCAC
CMPK2	cytidine monophosphate (UMP-CMP) kinase 2	mouse	GTTTCCTCGGTGTAGGAGCTGTGT CTCGAAGCTGACTTCACATGCAAT
MYH11	smooth muscle myosin heavy chain v1&2	mouse	ATGAGGTGGTCGTGGAGTTG GCCTGAGAAGTATCGCTCCC
MAPK14	mitogen-activated protein kinase 14	mouse	ATAAGAGGATCACAGCAGCCCAAG GACAGAACAGAAACCAGGTGCTCA
PICALM	picalm	mouse	CCATTCCAAGCTTAAACCCTTTCC AGGCCACTGTTGGTTTGAGAAGTC
GZMA	granzyme a	mouse	TGCTGCCCACTGTAACGTG GGTAGGTGAAGGATAGCCACAT
GZMB	granzyme b	mouse	TGCTGCTAAAGCTGAAGAGTAAG CGTGTTTGAGTATTTGCCCAT
NPC1	Niemann Pick type 1	mouse	GCTGTGAGCTGTGGTCTGC CTCACTCGGCTTCCTTTGGTA
NPC2	Niemann Pick type 2	mouse	AGGACTGCGGCTCTAAGGT AGGCTCAGGAATAGGGAAGGG
CCR7	C-C chemokine receptor type-7	mouse	TGTACGAGTCGGTGTGCTTC GGTAGGTATCCGTCATGGTCTTG
VDR	vitamin D receptor	mouse	ACCCTGGTGACTTTGACCG GGCAATCTCCATTGAAGGGG
CD44	CD44	mouse	GTCTTCTTCCGGCTCTCCATGTAA ATCTCACATCCAATGGGACAAGGT

### Immunocytochemistry and (immuno)histochemistry

After isolation, FCMs and NFMs were adhered to coverslips to allow identification of cell type (general leukocyte (Giemsa staining), macrophage or SMC/fibroblast; antibodies as listed in Table 2) after use of a mouse-on-mouse immunodetection kit (BMK-2212, Vector, USA) as necessary. Lipid content was identified after staining with Oil-Red-O (Sigma) and nuclei were counterstained with haematoxylin. Paraffin embedded sections of formalin-fixed sponges or brachiocephalic arteries from ApoE null mice were examined for expression of the various proteins (antibodies listed in Table 2), as were coverslips from RAW or bone marrow-derived monocyte (BMDM) cultures (S1 File). Specimens were visualised using light or fluorescent microscopy after labelling primary antibodies or controls with the appropriate secondary antibodies and substrate/fluorophore systems (DAB (Sigma), streptavidin-594 (Molecular Probes, Life Technologies). Nuclei were stained with haematoxylin (light microscopy) or DAPI (fluorescent; Prolong-Gold anti-fade medium + DAPI, Invitrogen). Irrelevant antibodies or appropriate sera were used in negative controls.

**Table 2:** Primary and secondary antibodies used in this study.

Antibody	Clone/Cat #	Type	Supplier
Primary antibodies/controls			
mouse monocyte/macrophage	MOMA2 /MBS530837	Rt_Mab	Biosource International, USA
Human α/γ-SM actin	HHF35 /M0635	Mm_Mab	Dako, UK
human smooth muscle myosin heavy chain (SM-1, SM-2)	hSM-V /M7786	Mm_Mab	Sigma, USA
human biglycan	- /ab58562	Gt_Pab	Abcam, USA
mouse CTGF	- /ab6992	Rb_Pab	Abcam
human LXRα (NR1H3)	- /LS-B3526	Rb_Pab	Lifespan Biosciences, USA
human cFOS	- /ab7963	Rb_Pab	Abcam
human phospho-SMAD2 (ser465/467)	138D4 /3108	Rb_Mab	Cell Signalling Technology (New England Biolabs, UK)
human total SMAD2	D43B4 /5339	Rb_Mab	Cell Signalling Technology
human phospho-SMAD3 (ser423/425)	C25A9 /9520	Rb_Mab	Cell Signalling Technology
human total SMAD3	C67H9 /9523	Rb_Mab	Cell Signalling Technology
rabbit GAPDH	6C5 /MAB374	Mm_Mab	Chemicon (Millipore)
normal rabbit IgG control	- /X0936		Dako
	- I5006		Sigma
normal rat IgG control	- /6-001-A		R&D Systems
normal mouse IgG1 control	- /X0931		Dako
normal mouse IgG2b control	- /X0944		Dako
goat serum	- /X0907		Dako
Secondary antibodies			
Mouse Ig	- /P0260	Rb_Pab	Dako
	- /B7264	Gt_Pab	Sigma
Rat Ig	- /E0468	Rb_Pab	Dako
	- /80-9520	Mm_Pab	Zymed (Invitrogen)
Rabbit Ig	- /P0448	Gt_Pab	Dako
	- /A0545	Gt_Pab	Sigma
	- /7074	Gt_Pab	Cell Signalling Technology
Goat Ig	- /E0466	Rb_Pab	Dako
	- /P0449	Rb_Pab	Dako

## Statistics

Statistical analysis of the microarrays was performed using the 'R' Bioconductor Lumi and Limma packages, applying the linear models and empirical Bayes methods included in the package [25]. Variance stabilizing transformation was performed using the Robust Spline Normalization (RSN) algorithm [26] with unsupervised analysis methods such as Principal Component Analysis and Hierarchical Clustering used for initial data exploration. Statistical analysis of differential expression consisted of a univariate model to detect individual genes that are significantly different in abundance between the conditions [25]. P-values were adjusted for multiple comparisons, using the False Discovery Rate (FDR) Benjamini-Hochberg method. Differential expression was classified as significant (P<0.01 after FDR correction for multiple testing) or suggestive (P<0.01 unadjusted). The significance of the association between the data set and the canonical pathway was measured by either a ratio of the number of molecules from the data set that map to the pathway divided by the total number of molecules that map to the canonical pathway is displayed, or by using a right-tailed Fisher’s exact test, to calculate a P-value determining the probability that the association between the genes in the dataset and the canonical pathway is explained by chance alone.

For the RT-qPCR, data differences between the two groups were tested for significance with Student’s *t*-test, using a logarithmic transformation or the Mann-Whitney U-test if the data were not normally distributed, using GraphPad Instat (USA). P-values of less than 0.05 were considered statistically significant. Values are expressed as mean ± standard deviation.

## Results

### Yield and purity of cells isolated from subcutaneous granulomas

**Fig 1:**
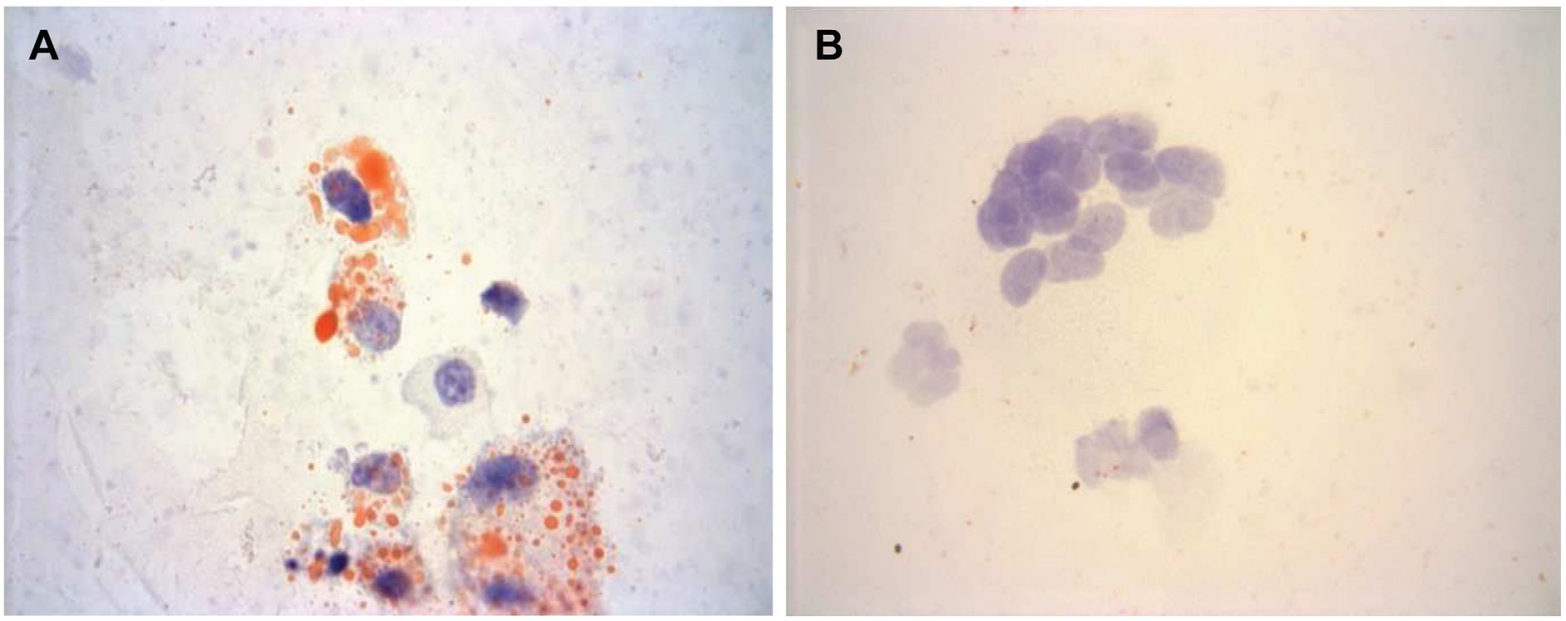
Lipid droplets were found only in macrophages from mice fed a high-fat diet. Oil-Red-O staining for lipid (orange-red) in A) FCMs and B) NFMs isolated from sponges. Nuclei appear purple (haematoxylin). Magnification x 400.

The yield of cells purified by floatation and adhesion from sponges implanted for 4 weeks into fat-fed ApoE null mice was 4.6 ± 2.7 x 10^6^. This was comparable to the 6.3 ± 2.4 x 10^6^ cells isolated by adhesion only from control non-fat-fed wild-type mice after 4 weeks, which is similar to values previously reported after 7 days [27]. Cells from the ApoE null and control mice were 96 ± 5.8% or 87 ± 10% MOMA2 positive, respectively, and only 0.86 ± 1.2% or 1.4 ± 1.8% α/γ-SM actin positive, respectively. Data from RT-qPCR confirmed that the cell preparations contained low levels of SM myosin heavy chain (SM1/SM2) (averaging less than 2.5 copies/ng RNA), implying they have little SMC contamination. Whereas almost all of the cells in the preparations from the ApoE null mice had lipid-rich (Oil-Red-O positive) inclusions, there were none in cell preparations from control mice (Fig 1). Hence cells isolated from the fat-fed ApoE null mice were indeed foam cell macrophages (FCMs), whereas those from the control mice were non-foamy macrophages (NFMs).

### Genes and pathways indicated from the array

**Fig 2:**
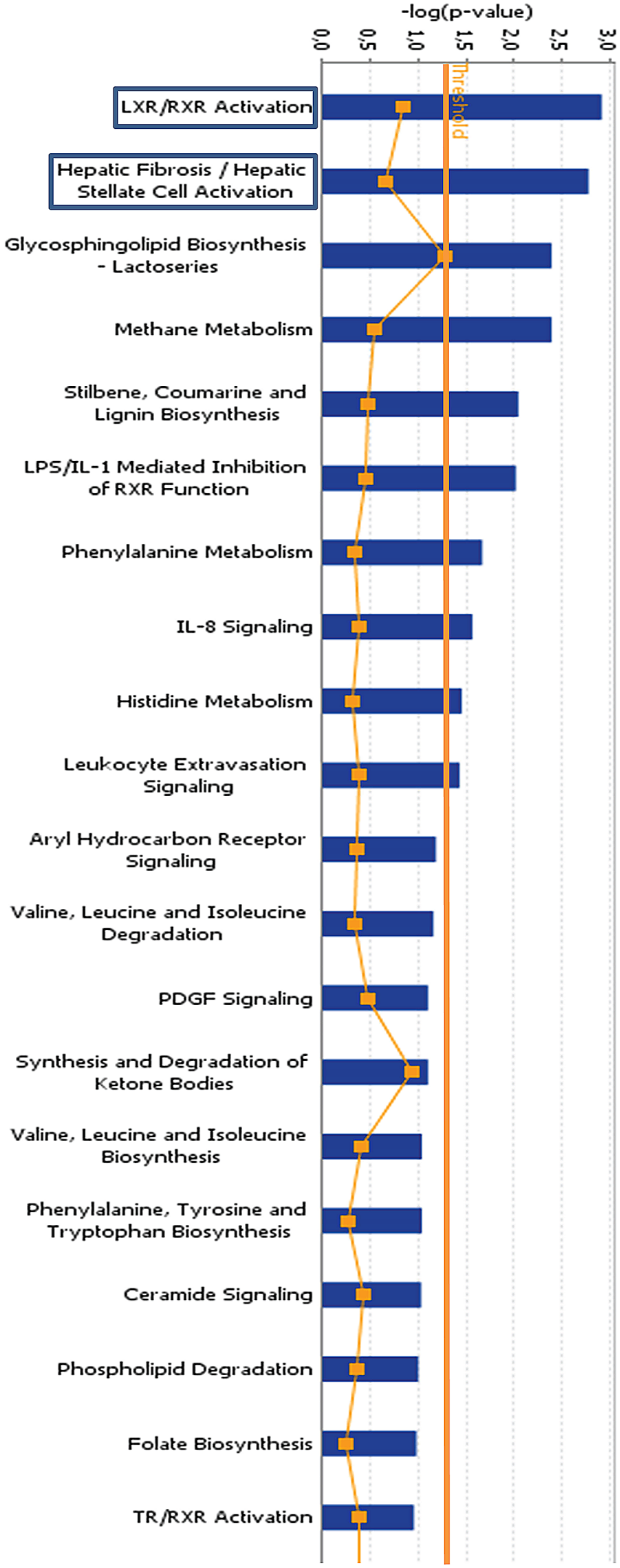
Canonical pathway analysis. The canonical pathways most enriched in FCMs were anti-inflammatory (LXR) and pro-fibrotic (derived from array data). Blue bars indicate significance, orange line indicates ratio. (Ingenuity Systems Inc).

RNA from FCMs and NFMs (n = 4) was subjected to microarray analysis on the Illumina platform (GEO accession number GSE70126). Using an adjusted P-value of <0.01, we found only 29 differentially expressed genes. However, using a less stringent unadjusted P-value <0.01, a total of 749 genes appeared to be differentially expressed, with 369 up-regulated and 380 down-regulated. A detailed list can be found in S1 Table. Canonical signalling analysis revealed that the most enriched pathway in FCMs compared to NFMs was that related to LXR and its obligate partner, retinoid acid receptor (RXR) (Fig 2), confirming the results of a previous study comparing peritoneal FCMs and NFMs from LDL receptor null mice [23]. The second most over-enriched pathway was that previously associated with hepatic fibrosis (Fig 2). Interestingly, functions found to be enriched and/or up-regulated in FCM by Ingenuity Functional Pathway analysis (Table 3) also implicated connective tissue development as well as cell regulation, differentiation and proliferation.

**Table 3:** Functions enriched/regulated in FCMs by Ingenuity Functional Pathway analysis.

Rank	Functions enriched/regulated in FCMs	Score
1	Dermatological diseases and conditions, Connective tissue development and function, Tissue morphology	52
2	Cancer, Cellular growth and proliferation, Nervous system development and function	30
3	Cardiovascular disease, Cellular growth and proliferation, Haematological system development and function	28
4	Cancer, Cellular movement, Cell death	23
5	Cell signalling, Molecular transport, Vitamin and mineral metabolism	23
6	Cellular growth and proliferation, Cancer, Cell death	13

**Fig 3:**
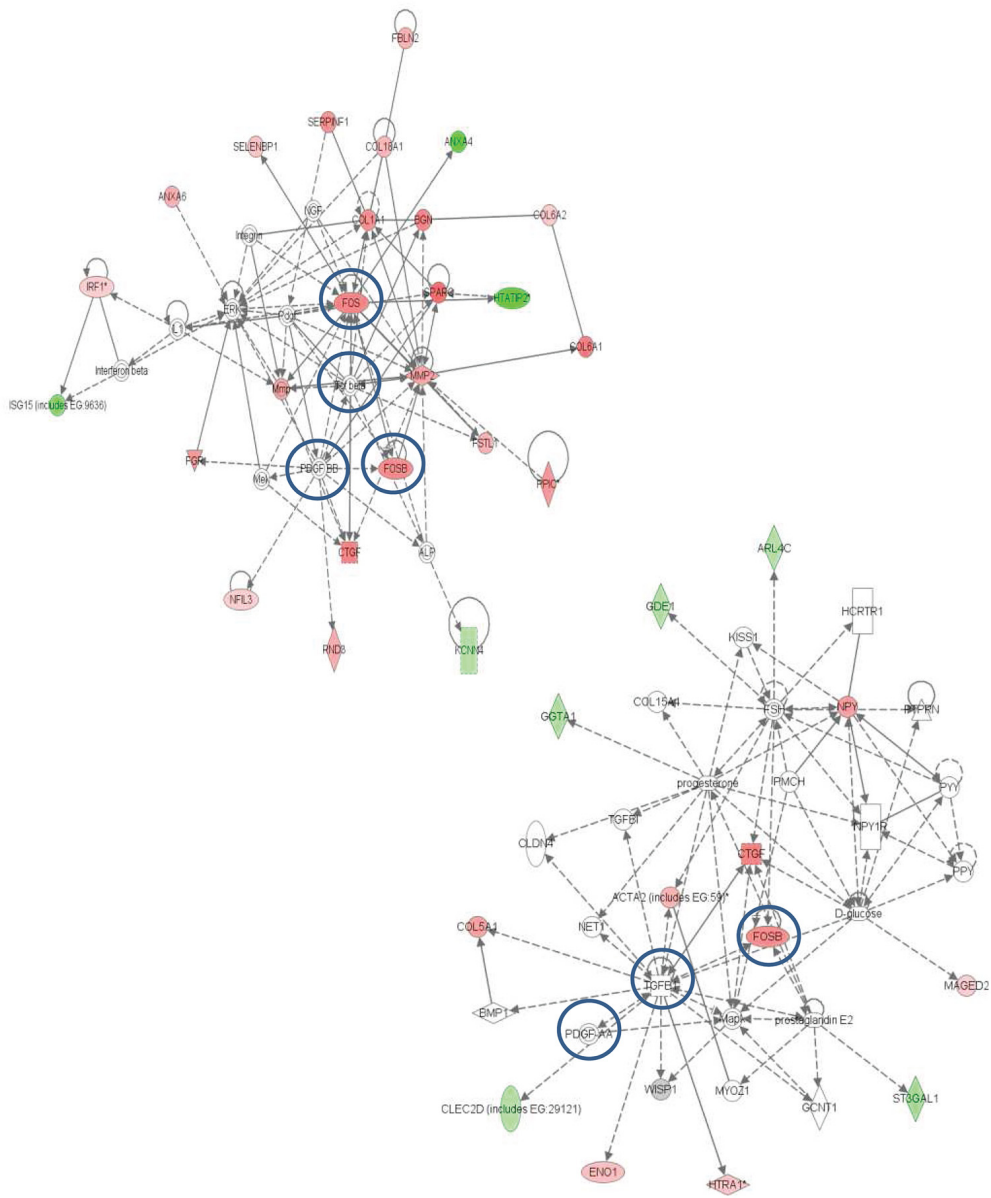
Network maps of genes differentially regulated in FCM and NFM array (top 2 networks). Molecules are represented as nodes, and the biological relationship between two nodes is represented as an edge (line). Continuous lines represent direct interactions, while indirect ones are represented by interrupted lines. The intensity of the node colour indicates the degree of up- (red) or down- (green) regulation. Colour of node indicates the presence (grey) or absence (white) of a given gene in the study. No change in M1/M2 markers were observed, but there was a regulatory node around PDGF and TGFβ (which were absent from the array) (Ingenuity Systems Inc).

The 2 most statistically significant networks generated from the array data (Fig 3) pointed to a regulatory node around platelet derived growth factor (PDGF) and transforming growth factor-β (TGFβ), which have been previously implicated in tissue fibrosis [28]. Network analysis also suggested that FOS and FosB, which are components of the activator protein-1 (AP-1) transcription factor complex that is known to be downstream of PDGF and TGFβ activation [29], might be major contributors to the differences between FCMs and NFMs (Fig 3). The network analysis focused our attention on extracellular matrix protein and pro-fibrotic genes, including several collagen polypeptides, decorin, biglycan and bone morphogenic protein 1 (BMP1, a pro-collagen convertase), that were strongly over expressed in FCMs relative to NFMs. We also further investigated expression of connective tissue growth factor (CTGF), which is a major contributor to fibrosis; and proteases, including cathepsins and matrix metalloproteinases (MMPs), which have been implicated in plaque rupture [30].

### Comparison of array and RT-qPCR measurements of differentially expressed mRNAs

**Fig 4:**
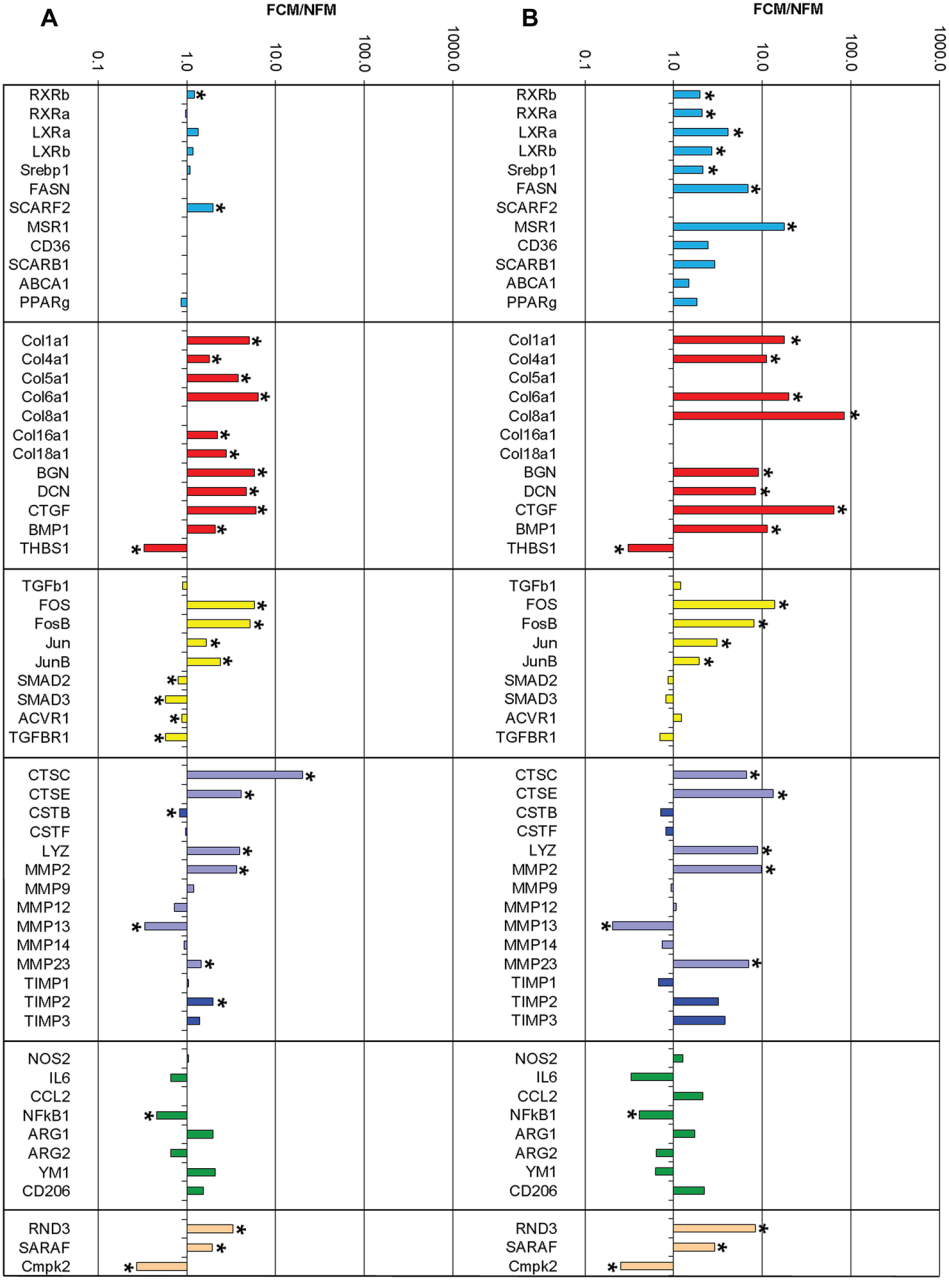
Genes differentially regulated in FCM and NFM. A) array; B) RT-qPCR). Cyan = LXR-related genes, red = extracellular matrix structural proteins, yellow = fibrosis-related signalling and associated molecules, purple/blue = degradative enzymes/inhibitors, green = genes associated with M1 and M2 polarisation, peach = miscellaneous genes. Genes not present on the Illumina chip or not qPCR verified are left blank. *P<0.05.

Despite the low stringency and hence potentially high false discovery rate of our initial analysis, most of the genes found to be differentially expressed in the array (Fig 4A) were validated by RT-qPCR, often with increased ratios (Fig 4B and Table 4). In addition, several candidate genes related to the identified pathways were found to be differentially expressed by RT-qPCR on greater numbers of preparations (Fig 4B and Table 4).

**Table 4:** Levels of mRNAs by RT-qPCR in FCMs and NFMs (n = 5–7).

Gene	FCM/NFM	FCMs (copies/ng RNA)	FCM SD	NFMs (copies/ng RNA)	NFM SD	P-value
36B4	1.39	3564	683.1	2558	228.0	0.3434
RXRβ	1.98	5903	3100	2982	1173	0.0303
RXRα	2.08	167.1	59.10	80.25	32.69	0.0170
LXRα	4.10	23.37	18.822	5.703	2.298	0.0177
LXRβ	2.69	2.000	1.219	0.743	0.108	0.0478
Srebp1	2.13	1332	712.8	624.3	174.7	0.0303
FASN	6.90	57.23	60.56	8.297	4.795	0.0182
MSR1	17.5	37074	45725	2116	1002	0.0038
CD36	2.47	1912	1890	774.8	880.3	0.4318
SCARB1	2.92	150.7	117.6	51.51	25.46	0.1061
ABCA1	1.48	44043	27951	29749	14210	0.3222
PPARγ	1.83	248580	95420	136141	30771	>0.9999
Col1α1	17.8	18252	14723	1024	708.9	0.0016
Col4α1	11.1	347.1	304.9	31.39	18.82	0.0041
Col6α1	19.7	1045	1194	52.95	41.89	0.0078
Col8α1	84.4	3.529	4.392	0.0418	0.0494	0.0380
BGN	9.08	22551	21086	2484	1678	0.0185
DCN	8.30	9964	9985	1201	795	0.0482
hCTGF	63.5	3.951	2.933	0.0623	0.0637	0.0001
BMP1	11.4	544680	473325	47913	20134	0.0081
THBS1	0.29	23317	7268	81297	37646	0.0051
TGFβ1	1.19	37.65	20.84	31.52	14.56	0.5855
FOS	13.7	29675	21365	2171	1416	0.0038
FosB	8.02	128.4	77.37	16.00	5.779	0.0001
JUN	3.09	24095	11387	7793	7144	0.0185
JunB	1.95	186454	63475	95703	51901	0.0255
SMAD2	0.87	946	585	1091	350	0.6325
SMAD3	0.82	132.5	100.0	161.1	105.1	0.6421
ACVR1	1.21	84.40	52.27	69.62	25.40	0.5750
TGFBR1	0.70	972	382	1392	1174	0.3101
CTSC	6.60	4003	2215	606.5	246.2	0.0033
CTSE	13.4	22.72	10.60	1.6993	1.1338	<0.0001
CSTB	0.72	27327	11840	38125	14139	0.1805
CSTF	0.82	235.1	127.5	285.1	136.3	0.5296
LYZ	8.84	105455	82637	11936	14926	0.0177
MMP2	9.82	654.4	500.6	66.62	39.62	0.0103
MMP9	0.94	799.2	562.8	854.6	823.8	0.8920
MMP12	1.07	13415	8590	12553	9439	0.8804
MMP13	0.20	2330	1452	11368	4487	0.0007
MMP14	0.75	35000	12138	46875	25061	0.4901
MMP23	6.98	5109	3772	732.0	388.9	0.0080
TIMP1	0.67	2037	1393	3023	1707	0.2955
TIMP2	3.23	692.0	637.5	214.5	148.5	0.2677
TIMP3	3.81	19.70	18.27	5.165	5.289	0.2677
NOS2	1.27	13143	8979	10331	5791	0.5550
IL6	0.33	443.6	467.4	1333	1419	0.2126
CCL2	2.15	3.290	2.290	1.530	0.850	0.1061
NF-κB1	0.41	978.9	405.6	2378	1248	0.0219
ARG1	1.71	72449	55730	42307	45624	0.3862
ARG2	0.63	6887	4015	10866	6745	0.2270
YM1	0.63	1198	610.1	1905	891	0.1315
CD206	2.22	34553	14498	15565	5164	0.5934
RND3	8.43	104.32	54.87	12.38	6.575	0.0004
SARAF	2.88	996.1	710.6	345.3	147.0	0.0194
Cmpk2	0.25	1265	895	5010	2654	0.0129

#### LXR-related genes

Genes associated with the LXR pathway that was identified by canonical signalling analysis were of special interest, given the previous data comparing peritoneal FCMs and NFMs from LDL receptor null mice [23]. RXRβ was increased 1.2-fold in the array (P = 0.0019, Fig 4A) and this was confirmed by RT-qPCR (2-fold, P = 0.0303) with over 1000 copies/ng RNA in FCMs (Fig 4B and Table 4). RXRα was also over expressed in FCMs (P = 0.0170) but these cells only had 160 copies/ng RNA. LXRα and LXRβ were over expressed in FCM (P = 0.0177 and P = 0.0478, respectively), although both had less than 25 copies/ng RNA. Looking at downstream genes, sterol regulatory element binding protein 1 (Srebp1) was increased in FCMs (P = 0.0303) and had over 1000 copies/ng RNA, whereas fatty acid synthase (FASN), also increased in FCMs (P = 0.0182), had less than 60 copies/ng RNA. Scavenger receptor class F member 2 (SCARF2) was 2-fold elevated in FCMs in the array (P = 0.0081) and macrophage scavenger receptor 1 (MSR1, scavenger receptor A1 (SR-A1)) was increased 17.5-fold in FCMs by RT-qPCR (P = 0.0038). The mRNAs for scavenger receptors CD36 and scavenger receptor class B, member 1 (SCARB1, SR-B1) (both associated with TGFβ [28,31–34]), although abundant in FCMs, were not significantly different from those in NFMs. There were no significant differences between FCMs and NFMs in ATP binding cassette transporter isoform A1 (ABCA1) or peroxisome proliferator-activated receptor-γ (PPARγ expression, although both FCMs and NFMs had very high expression levels of these genes (130000–250000 copies/ng RNA), as previously reported [10].

#### Extracellular matrix structural proteins

Probably the most unexpected finding from our experiments was that several collagen polypeptide genes, as well as core proteins of the proteoglycans, biglycan and decorin, and the matricellular protein CTGF, were 2 to 6.5-fold up-regulated in FCM compared with NFM in the array (Fig 4A). All of these genes are known targets for induction by PDGF and/or TGFβ [29,35]. The large increases in mRNAs for collagens 1, 4 and 6 were confirmed by RT-qPCR (11–20 fold increase in FCM, Fig 4B and Table 4). Collagen1α1 was the most abundant, with 18000 copies/ng RNA in FCMs and 1000 in NFMs (P = 0.0016, Table 4). FCMs had more than 63-fold more mRNA for CTGF than did NFM (P = 0.0001), also confirming the array result. The mRNAs for biglycan and decorin core proteins were also significantly increased and highly abundant, with 23000 or 100000 copies in FCM and only 2900 or 1200 in NFM, respectively (biglycan P = 0.0185, decorin P = 0.0482). The expression of BMP1, which is known to be up-regulated by TFGβ and potentially involved in the regulation of TGFβ activation [36], was considerably increased in FCM, with 540000 and 48000 copies/ng RNA in FCMs and NFMs, respectively (P = 0.0081; Fig 4B and Table 4). By contrast, thrombospondin 1 (THBS1), which is known to bind and activate TGFβ [36] was significantly reduced in FCM (P = 0.0051; Fig 4B), although both FCMs and NFMs had abundant THBS1 mRNA (23000 and 81000 copies/ng, respectively, Table 4).

#### Fibrosis-related signalling and associated molecules

Levels of TGFβ1 mRNA were similar in FCMs and NFMs (Fig 4B). However, the PDGF and TGFβ related activator protein-1 (AP-1) family members FOS and FosB were over expressed over 5-fold, whereas JUN and JunB were 1.5 and 2.4 fold higher, respectively, in FCMs compared to NFMs in the array (Fig 4A). Data from RT-qPCR (Fig 4B) confirmed that FCMs expressed more FOS, JUN and JunB than NFMs, with several thousand copies of each per ng RNA (Table 4). The level of FosB expression was lower than the other signalling molecules, but still over-expressed in FCMs compared with NFMs, with 130 copies/ng RNA in FCM and only 16 copies/ng RNA in NFM (P = 0.0001; Fig 4B). By contrast with the increases in AP-1 factors, signalling factors SMAD 2 and 3 that are activated by TGF family members by phosphorylation appeared to be down-regulated in FCMs in the array, as were the TGFβ receptors activin A receptor type 1 (ACVR1, ALK2) and TGFβ Receptor 1 (TGFBR1, ALK5) (Fig 4A). However, none of these small fold changes could be confirmed by RT-qPCR (Fig 4B).

#### Degradative enzymes

The most differentially expressed gene in the array was cathepsin C (CTSC), with a 20-fold change between the FCM and the NFM (P = 0.0000, Fig 4A). This difference was confirmed by RT-qPCR (Fig 4B) with 4000 and 600 copies in FCMs and NFMs, respectively (Table 4). Cathepsin E (CTSE) was also over expressed in FCMs (Fig 4A and 4B), although there were few copies of mRNA in either type of macrophage. In contrast, cystatins E and F were either not changed or slightly decreased in FCMs (Fig 4A and 4B and Table 4). Lysozyme (LYZ) was over-expressed (4-fold) in FCMs in the array (Fig 4A) and RT-qPCR (Fig 4B), where FCMs and NFMs contained 105000 and 12000 lysozyme copies/ng RNA, respectively (P = 0.0177, Table 4). Turning to the MMPs, we found that the gelatinase, MMP2, and the cysteine-rich MMP, MMP23, were over expressed in FCMs (650 or 5100 copies in FCM and only 67 or 730 in NFM, respectively). In contrast, for those MMPs that are related to M1 activation [24], there was no change in MMP9 (Table 4) and the abundant M1-related collagenase, MMP13, was down-regulated in FCMs (2.9-fold, P = 0.0302 in the array and 4.9 fold P = 0.0007 by RT-qPCR). The tissue inhibitor of MMPs, TIMP-2, appeared to be increased in FCMs compared with NFMs in the array, although this could not be confirmed using RT-qPCR (Fig 4A and 4B and Table 4).

#### Genes associated with M1 and M2 polarization

From the array, there were no differences in genes associated with M1 macrophage activation, such as inducible nitric oxide synthase (NOS2), IL-6, MMP9 or chemokine (C-C motif) ligand 2 (CCL2, MCP1). However, as mentioned above, MMP13 was decreased, and in the NF-κB pathway, which is implicated in M1 activation [37,38], NF-κB_1_ was also down-regulated in FCMs in the array (Fig 4A). Data from RT-qPCR (Fig 4B), confirmed that NOS2 expression was high in both FCM and NFM, 13000 and 10000 copies/ng RNA, respectively (P = 0.5550). CCL2 levels were also not significantly different in FCMs and NFMs (P = 0.1061), nor was MMP9 (Table 4). NF-κB_1_ was lower in FCM than in NFM, with 980 and 2400 copies/ng RNA in FCMs and NFMs, respectively (P = 0.0219, Fig 4B and Table 4), confirming the array data. There were no differences in M2 marker genes, Arg1, Ym1 and the mannose receptor (CD206) in the array (Fig 4A). By RT-qPCR, Arg1 expression in both FCMs and NFMs was high, with 72450 and 42300 copies/ng RNA, respectively (P = 0.3862). Although Arg2 levels (suggesting either M1 or M2c activation) were lower in the FCM, the differences did not reach significance (FCM 6900, NFM 11000 copies/ng RNA, respectively, P = 0.2270). Both FCMs and NFMs expressed the M2 markers Ym1 (1000–2000 copies/ng RNA) and CD206 (15000–35000 copies/ng RNA) at similar levels. Together these results indicate that FCMs and NFMs display similar levels of established markers for either M1 or M2 polarization.

#### Miscellaneous genes

Other genes that were confirmed as differentially regulated in the two set of macrophages were Rho family GTPase 3 (RND3), which regulates the organisation of the actin cytoskeleton in macrophages and other cells [39] and store-operated calcium entry-associated regulatory factor (SARAF, formerly Tmem66); both were elevated in FCMs (P = 0.0004 and P = 0.0194, respectively). Cytidine monophosphate (UMP-CMP) kinase 2 (Cmpk2), which is involved in the terminal differentiation of monocytic cells [40], was lower in FCMs (P = 0.0129, Fig 4B). Further details of the differences in the differentially expressed genes that were validated using RT-qPCR can be found in Table 4.

### Immunohistochemical validation of selected findings from the array

Given that the two pathways most increased at the mRNA levels in FCMs compared to NFMs were the LXR and hepatic fibrosis pathways (Fig 2), we used immunohistochemistry to validate these findings in sections of subcutaneous sponges from ApoE null mice. We also took the opportunity, where possible, to compare plaque FCMs and adventitial NFMs in the same atherosclerotic plaques from the brachiocephalic artery of ApoE null mice.

#### LXRα

**Fig 5:**
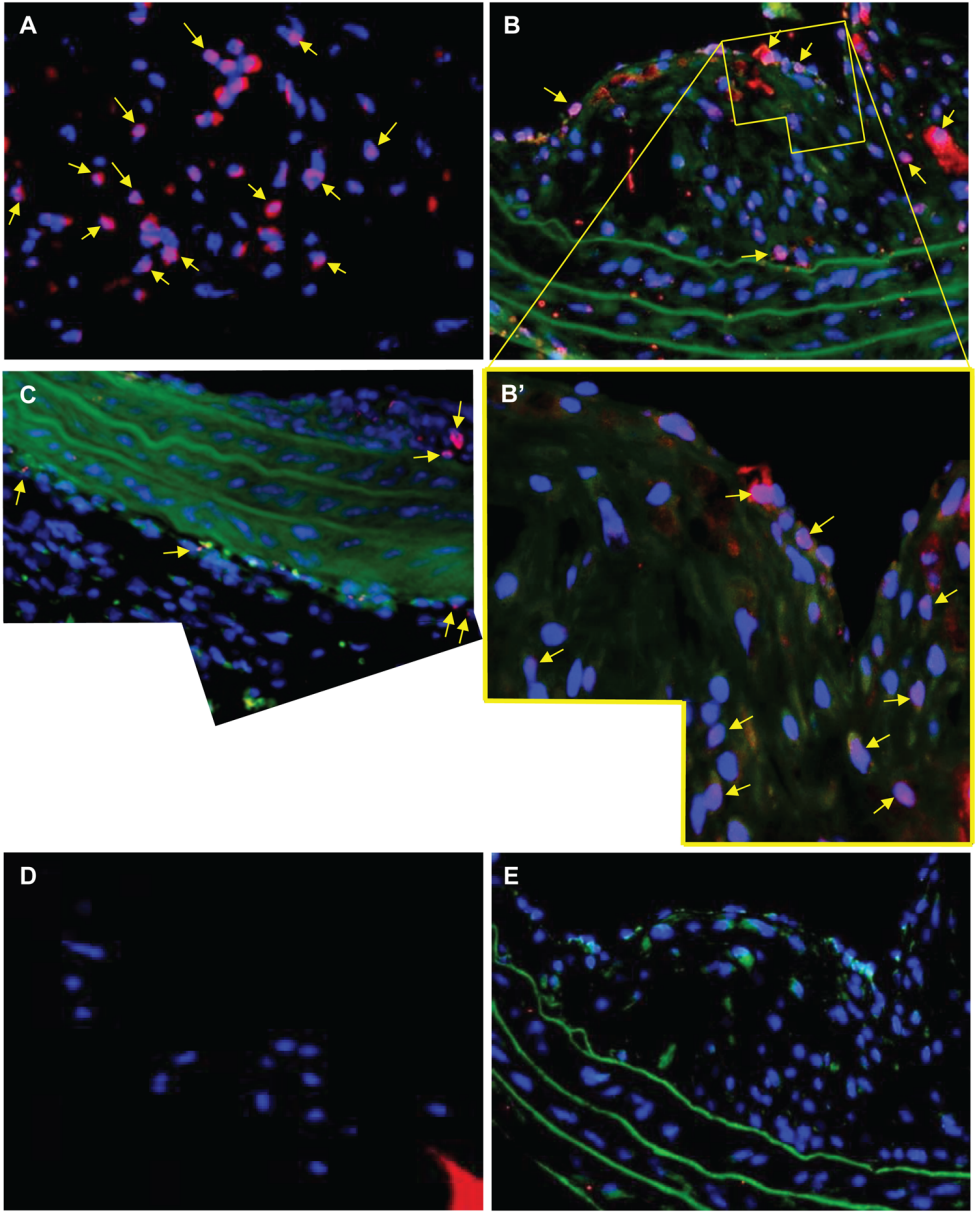
LXRα in sponges and arteries from fat-fed ApoE null mice. LXRα is present in the cytoplasm (red) and/or nucleus (pink, arrows) of A) FCMs in sections from a subcutaneous sponge, or; B) cells in the plaque of a brachiocephalic artery. B’ higher magnification of plaque in B. C) LXRα is occasionally present in the cytoplasm of the adventitial cells that are close to the media; D) sponge section negative control (only the sponge spicule is red); E) negative control in a section from the same plaque as B. Blue = nuclei (DAPI), green = autofluorescence. Magnification x 400 A-E, x 1000 B’.

LXRα protein was identified in 52 ± 26% of cells (n = 5) in sections of sponges from ApoE null mice (Fig 5A) and in 53 ± 23% of the cells in plaques from brachiocephalic arteries from ApoE null mice (Fig 5B, 5B’ and 5C,). Cells with nuclear (pink) and cytoplasmic (red) staining were found throughout the plaque. Cells containing LXRα in their cytoplasm were most often found near the lumen, while those cells closest to the internal elastic lamina, deep within the plaque, tended to have less cytoplasmic LXRα (Fig 5B). Little cytoplasmic (and even less nuclear) staining was found in adventitial cells (Fig 5C), and no staining at all in IgG control sections (Fig 5D and 5E).

#### CTGF

**Fig 6:**
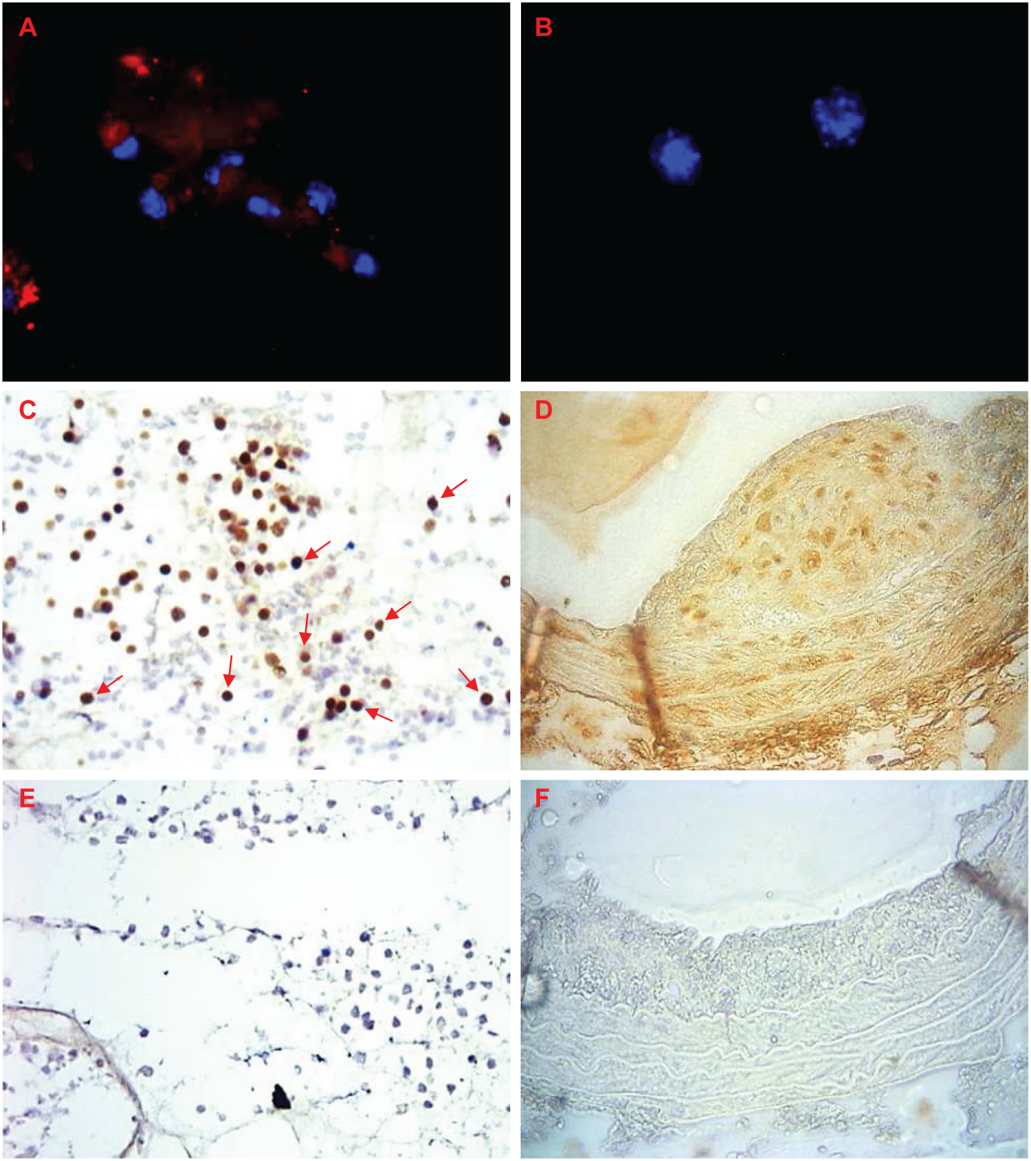
CTGF in sponges and arteries from mice. CTGF (red) is present in A) in FCMs, but not B) NFMs isolated from sponges. Blue = nuclei (DAPI). CTGF (brown) is present in C) FCMs in sponge sections or; D) throughout the plaque, media and adventitia of a brachiocephalic artery from a fat-fed ApoE null mouse. E) sponge section negative control; F) negative control in a section from a brachiocephalic artery plaque. Magnification x 200 (C, E), x 400 (A, B, D, F).

Most FCMs but very few NFMs isolated from sponges stained for CTGF (Fig 6A and 6B; red staining), which supports the mRNA data. Of cells in sponge sections from fat-fed ApoE null mice, 49 ± 22% (n = 10) stained for CTGF (arrows in Fig 6C). FCMs in brachiocephalic artery plaque sections also had strong staining for CTGF (Fig 6D). SMC in the media also stained for CTGF, as did the extracellular matrix of the adventitia, which obscured any staining by adventitial cells. Approximately 90–95% (n = 6–7) of cells in the plaque, media and adventitia stained for CTGF protein, intracellularly or in the surrounding matrix. This data therefore confirmed the over expression of CTGF in sponge and plaque FCMs, and suggested that there might be reduced staining from NFMs, at least in in the sponges.

#### cFOS

**Fig 7:**
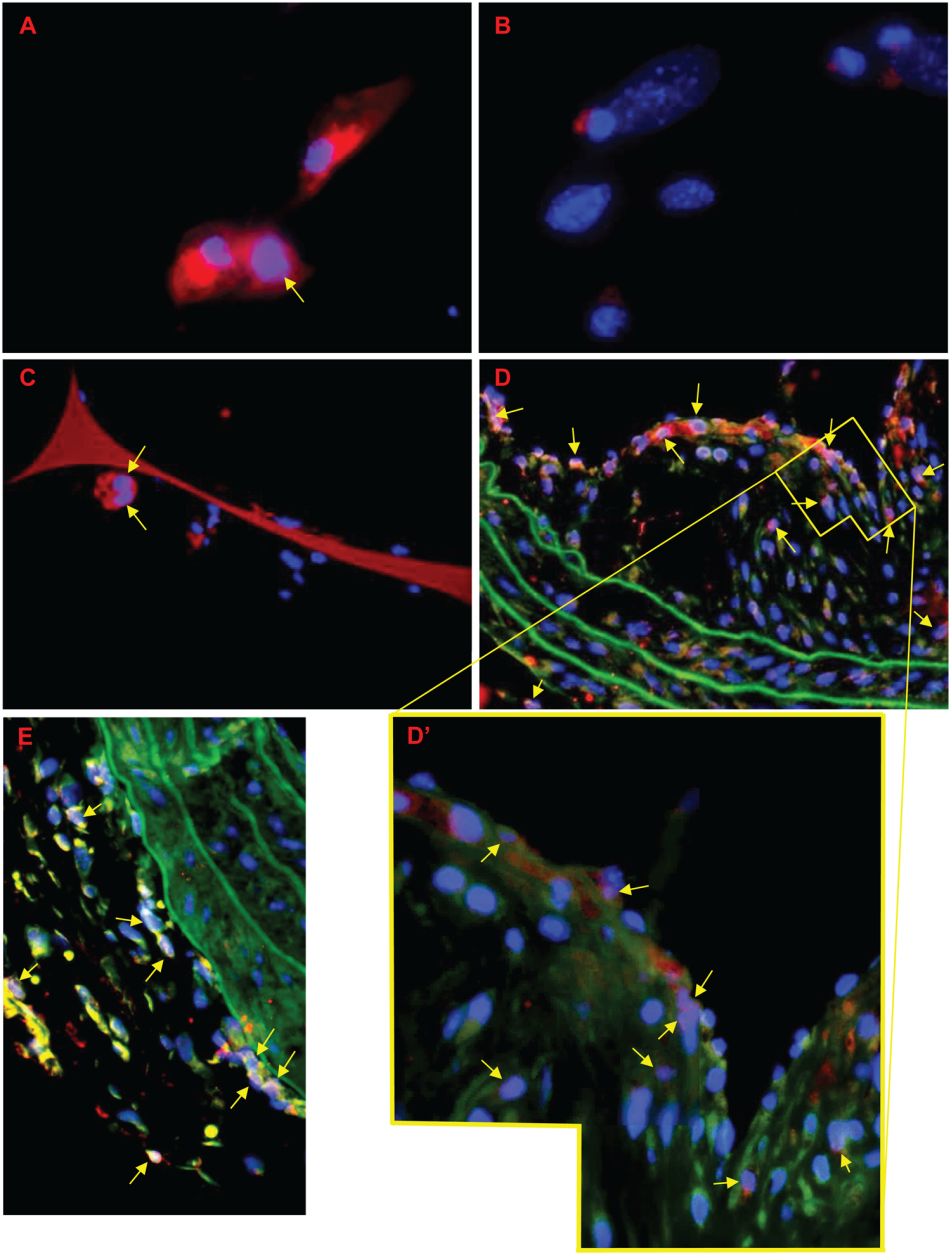
cFOS in sponge and artery macrophages from mice. cFOS is present in the cytoplasm (red) or nucleus (pink, arrows) in isolated macrophages from A) mice fed a high-fat diet (FCMs) or B) a normal diet (NFMs). cFOS was also observed in the cytoplasm (red, orange, yellow) and/or nucleus (pink, arrows) of cells in sections from C) a subcutaneous sponge granuloma or D, E) a brachiocephalic artery from a fat-fed ApoE null mouse. D’) higher magnification of plaque in D. Blue = nuclei (DAPI), green = autofluorescence. Magnification x 400 A-E, x 1000 D’. See Fig 5C and 5D for negative control staining.

FCMs isolated from sponges had prominent cFOS immunostaining in their cytoplasm (red), with a third of cells also expressing cFOS in their nucleus (pink) (Fig 7A). Staining was less pronounced in isolated NFMs (Fig 7B), and was only observed in a few nuclei. Staining for cFOS was also observed of FCMs in subcutaneous sponge granulomas (Fig 7C), with 42 ± 22% of cells (n = 5) having nuclear cFOS staining (pink), and many having cFOS staining (red) in their cytoplasm. 43–44% of cells within the BCA plaques (n = 2) also expressed nuclear (pink) cFOS, with many also having cytoplasmic cFOS staining (Fig 7D and 7D’). A similar proportion, 45–55%, of adventitial cells (from their shape possibly fibroblasts) also had nuclear cFOS staining (Fig 7D and 7E). Overall, almost half of all cells in the plaque, adventitia or sponge sections had nuclear cFOS staining. Clearly, at least some plaque FCMs were cFOS positive, which confirms the array and RT-qPCR findings.

#### Role of TGFβ1 and activation of SMAD2 signalling in FCMs

**Fig 8:**
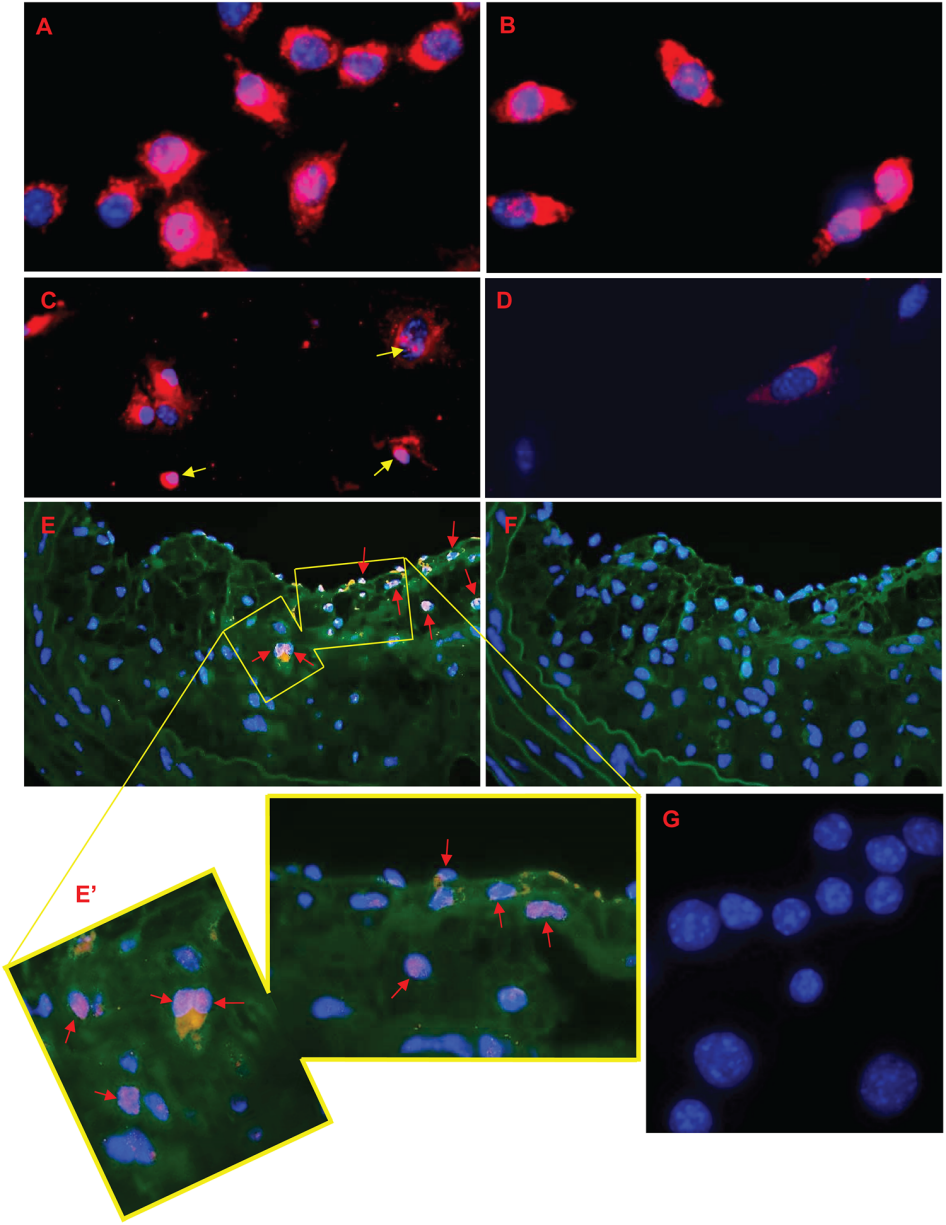
Phosphorylated SMAD proteins in RAW 264.7 cells and sponge or artery macrophages from mice. RAW cells were exposed to 10 ng/ml TGFβ1 for 45 minutes. A) phospho-SMAD2 and B) phospho-SMAD3 were present in the cytoplasm and nucleus of many cells. pSMAD2 was also found in C) the cytoplasm and nucleus (arrows) from isolated FCMs, but only in the D) cytoplasm of NFMs. E) pSMAD is present in the cytoplasm and nuclei (arrows) of plaques in a brachiocephalic artery a fat-fed ApoE null mouse. E’) higher magnification of plaque in E. F) negative control in a section from the same plaque. G) RAW negative control staining (rabbit IgG). Red/orange = cytoplasmic staining, pink = nuclear staining, blue = nuclei (DAPI), green = autofluorescence. A, B, E’, G magnification x 1000, C, D, E, F magnification x 400.

Members of the TGF family signal through phosphorylation and nuclear translocation of SMADs, especially SMAD2. Hence we hypothesised that FCMs in sponges and plaques might contain increased levels of nuclear pSMAD2 detectable by immunohisto/cytochemistry. As a positive control, we first showed that TFGβ1 quickly stimulated SMAD2 and SMAD3 phosphorylation and translocation to the nucleus by 45 minutes in mouse RAW cells (Fig 8A and 8B and Table B in S1 File). Staining for nuclear pSMAD2 (pink) was found in 50 ± 12% of isolated FCMs; many also had cytoplasmic staining (red). By contrast, nuclear pSMAD2 staining was detected in only 10 ± 10% of NFM (n = 3, P = 0.0110, Fig 8C and 8D). Furthermore, 74–76% of the FCMs in brachiocephalic artery plaques had pSMAD2 present in their nuclei (pink), with many also having pSMAD2 in their cytoplasm (orange) (Fig 8E and 8E’). No staining was observed in an IgG control section from the same plaque or in IgG control RAW cells (Fig 8F and 8G). This provides strong evidence for SMAD2 signalling in FCMs in sponges and plaques.

## Discussion

### Main findings

Our experiments document novel differences between the transcriptomes of FCMs and NFMs obtained from subcutaneous sponge-induced granulomas in living ApoE null mice. The most significant changes were in the LXR/RXR pathway, which concurs with a study of peritoneal FCMs and NFMs in LDL receptor null mice [23]. We also observed significant increases in fibrosis-related gene expression in FCMs, including several collagen polypeptides and proteoglycan core proteins. Furthermore, we observed elevated levels of FOS and JUN transcription factors that are associated with pro-fibrotic actions [41] and phosphorylation of the SMAD2 transcription factor that mediates actions of TGF family members [42]. FCMs from subcutaneous sponges from ApoE null mice were polarized neither towards the M1 (classically activated) nor M2 (alternatively activated) phenotypes. Our results demonstrate that FCMs formed *in vivo* adopt a pro-fibrotic phenotype.

### LXR/RXR pathway

LXRs act by heterodimer formation with RXRs and subsequent binding of the complex with LXR response elements in target genes. They can also inhibit expression of other genes by antagonising the activity of transcription factors or by preventing the release of co-repressor complexes from target-gene promoters [43]. We found that FCMs had increased expression of LXRα and LXRβ, as well of their binding partners RXRα and RXRβ, as well as several down-stream mediators (e.g. Srepb1, FASN). Similar observations were reported previously in peritoneal macrophages isolated from fat-fed LDL receptor null mice [23]; and loading of mouse BMDM with acetylated low density lipoprotein (LDL) *in vitro* also upregulates several LXR-related genes [44]. On the other hand, loading with oxidised- (ox-) LDL for 24 hours down-regulates MSR1, FASN and SCARB1 but upregulates CD36 and ABCA1, most likely by modulating transcription by ATF3 [45]. Such disparities dependent on both the type of lipids used and the phenotypic state of the mouse macrophages before loading have been previously reviewed [46].

### The pro-fibrotic response of FCMs

In addition to lipid-related genes, we found that the mRNAs of many extracellular matrix proteins were up-regulated in FCMs. These included mRNAs for collagens 1, 4, 5, 6, 8, 16 and 18 as well as the pro-collagen convertase, BMP-1. Furthermore, mRNAs for the core proteins of biglycan, decorin and versican, which are proteoglycans that bind to collagen and promote matrix assembly and maturation, were elevated in FCMs (Table 4 and S1 Table). All of these matrix proteins are present at high levels within plaques [47–49] but have been thought of as products of SMCs or fibroblasts [35]. Our new data suggests that FCMs contribute to their own surrounding extracellular matrix. Interestingly, FCMs recovered from the peritoneum of LDL receptor null mice also overexpress collagen 1α2, collagen 3α1, collagen 6α1 and decorin by 2–4 fold compared to NFMs (Supplementary Table IC of [23]). Hence the pro-fibrotic transformation we observed in cells from subcutaneous granulomas in ApoE mice is replicated in the LDL receptor null background and at another site. On the other hand, several transcriptomic studies of mouse BMDM loaded with lipids for short periods *in vitro* did not note any changes in fibrosis-related genes [44,45,50], which suggests that such changes may evolve slowly or depend on the local microenvironment *in vivo*.

Collagens 1 and 3 are believed to be important in stabilizing plaques against rupture [51]. In contrast, collagen 8 coincides with active remodelling, migration and increased production of some MMPs [52,53]. Biglycan and versican are known to trap lipid within the matrix and hence perpetuate lesion progression leading ultimately to instability [48,49,54]. The impact of fibrotic transformation of FCMs could therefore be beneficial for stability through collagen synthesis but promote plaque progression through proteoglycans.

Canonical pathway analysis and network maps suggested PDGF and TGFβ as a regulatory node in the gene expression changes in FCMs compared to NFMs (Fig 3). At first sight this seems counter-intuitive, because TGFβ has been shown to reduce foam cell formation *in vitro* by decreasing scavenger receptor mRNA expression and oxidised LDL uptake [4]. However, much previous literature establishes that TGFβ can stimulate collagen synthesis in the plaques [29,35], as well as during the development of restenosis [55]. Proteolytic activation of latent TGFβ, rather than increased expression, is often responsible for increased TGFβ activity [29]. Consistent with this, we found that TGFβ1 mRNA was not increased in FCMs compared with NFMs; nor was the TGFβ1 receptor, ALK5. However, MMP2 was increased; and BMP1, which can also activate TGFβ, was up-regulated 11-fold in FCMs compared with NFMs. CTGF (CCN2), a multi-functional growth factor that is up-regulated by TGFβ via AP-1 and SMADs [28,56,57] was also increased in FCMs. CTGF may act synergistically with TGFβ [28,56] and can promote monocyte migration into the atherosclerotic plaque [58]. To validate our differential gene expression data *in vivo*, we showed that CTGF staining in both FCM- and SMC-rich areas of mouse plaques (Fig 6D). CTGF staining has also been reported in human atherosclerotic plaques, particularly near areas associated with large numbers of macrophages e.g. the shoulder region and surrounding the lipid core [58], and alveolar macrophages examined *in vivo* have been shown to express CTGF [59]. On the other hand, thrombospondin-1, which is also implicated in the binding and activation of TGFβ [31], was significantly decreased in FCMs.

TGFβ signalling is mediated by phosphorylation of SMADs, and by up-regulation of FOS, JUN and LXR transcription factors [28,34,36,55,60,61]. Consistent with this, we found that although the mRNA expression of SMADs 2 and 3 were not elevated in FCMs, levels of phosphorylated, nuclear localised SMAD2 were increased in granuloma FCMs relative to NFMs and in plaque FCMs, suggesting that TGFβ1 signalling was occurring. SMAD2 staining has been previously associated with FCMs in fibro-fatty lesions [60]. FCMs also had elevated expression of FosB, JUN and JunB. Interestingly, other factors that are affected by the presence of FosB, such as the extracellular matrix protein tenascin C [57], were also significantly up-regulated in our FCMs (S1 Table). We also confirmed that mouse FCMs overexpressed cFOS, with protein expression often found in the nucleus.

### Proteinases

Cathepsins C and E were overexpressed in *in vivo* generated FCMs in our study and also in mouse BMDM loaded with acetylated LDL *in vitro* [44]. However, these are acidic proteases, and only cathepsins with activity at neutral pH have been directly implicated in destabilization of plaques [62,63]. We found increased expression of MMP2, which promotes SMC migration and proliferation [64]. MMP2 is also known to activate latent TGFβ [65] and release TGFβ from extracellular matrix stores [55], which could further contribute to a pro-fibrotic action. MMP9, which also promotes migration of SMC [64], was not changed in our study, despite data from peritoneal FCMs showing a decrease [23]. Instead, we observed decreased expression of MMP13, which is the main collagenase of mouse atherosclerosis [66]. On the other hand, the expression of MMP23, which has not been studied in the context of atherosclerosis, was increased.

### Polarization towards M1 or M2 phenotypes

Peritoneal FCMs appeared to be polarized away from M1 compared to NFMs in LDL receptor null mice [23]. Characteristic M1 genes, including IL1β and MMP9, were down-regulated and, moreover, peritoneal FCMs were resistant to the M1 polarizing effects of added toll-like receptor ligands *ex vivo*, in part because accumulation of desmosterol led to ligation of LXR, which stabilized the co-repressor complex, NcoR [23]. In contrast with these results, we did not observe a significant decrease in M1 marker genes, including MMP9, NOS2, CCL2, IL-6 and ARG2, in granuloma FCMs compared with NFMs, although MMP13 and NF-κB1 levels, which are also M1-related genes [24,37,38], were decreased. There could be several reasons for this discrepancy, including different diets and background strains in the two studies and the metabolic consequences of ApoE compared with LDL receptor knockout. Another difference is that we took FCMs from subcutaneous granulomas rather than the peritoneum, and the cells therefore experienced a different inflammatory environment *in vivo*. NFMs from the peritoneum are known to be strongly polarized towards M2 [67], whereas, given the foreign body reaction in granulomas, it is not surprising that we measured considerable levels of M1 markers in both NFMs and FCMs (Table 4). Our previous work on subcutaneous granulomas in lipid- or chow-fed, wild-type rabbits also demonstrated the presence of M1 markers, but these were greater in FCMs than NFMs. FCMs had greater activation of NF-κB and up-regulation of the NF-κB dependent genes, MMP1 and MMP3; they also had decreased expression of arginase-1 and increased nitrite production compared with NFMs [21,22], confirming polarization away from M2. In the only available study of human plaque cells examined *ex vivo*, cells including FCMs also expressed M1 genes, and this was dependent on toll-like receptor-2 (TLR2) stimulation [68]. Hence M1/M2 polarisation appears to depend crucially on the microenvironment from which the FCMs are obtained. FCMs appear polarised towards M1 when extracted from human plaques [68] and rabbit granulomas [21,22], towards M2 when extracted from the peritoneum of LDL receptor null mice [23] or neither M1 nor M2 when extracted from mouse subcutaneous granulomas in this study. Consistent with this, FCMs bearing M1 markers, M2 markers or neither are detected in mouse plaques by histology [69] and FCMs observed histologically in human plaques also demonstrate a wide variety of phenotypes [9]. Many FCMs appear to be M1, based on nuclear NF-κB localisation and expression of marker genes such as NOS2 and COX2 [8,19]. Laser capture dissected plaque macrophages also show up-regulation of several M1-related genes, including NOS2, Arg2, TLR2 and IL1r1 [70]. However, FCMs bearing M2 markers such as CD206 and PPARγ [19] are also present, although they tend to be less foamy, probably thanks to up-regulation of reverse transporters [13]. In plaques, FCMs may also ingest particles other than LDLs, including bacteria, apoptotic bodies and cholesterol crystals that can cause additional polarization towards M1 [71,72], or take up haem and become deactivated [16].

A similar disparity of findings has been obtained in *in vitro* studies, depending on the type of lipid used (e.g. minimally compared to extensively oxidised LDL, acetylated LDL, oxidised phospholipids or cholesterol, cholesterol crystals) and the phenotypic state of the mouse macrophages before loading (reviewed by Adamson and Leitinger [46]). For example, ox-LDL loading of M2 macrophages generates a pro-inflammatory state [50], but oxidised phospholipid treatment leads to a distinct, anti-oxidant state [69]. Lipid ligands of PPARγ can prime macrophages towards an anti-inflammatory state [13]. In agreement with this diversity of responses, one study of ox-LDL loaded human monocyte derived macrophages observed overexpression of M1 genes thanks to toll-like receptor activation [20] but another found little effect on M1 or M2 markers, MMP-14 or TIMP-3, at least at the mRNA level [73], consistent with our present findings.

### Limitations of our study

Since FCMs and NFMs were both harvested from subcutaneous sponges in our study, the foreign body response to the sponge itself could not obscure the transcriptomic differences, including up-regulation of pro-fibrotic genes, which we observed in FCMs. However, we do not yet know how significantly these contribute to plaque progression and stability in man. Supporting the relevance of our present data in mice, formation of human FCMs *in vitro* has been previously shown to up-regulate expression of many of the same genes, including CTSC, LXR, Cmpk2, and fibrotic genes [14,17,18,40,74]. Studies in human and mouse plaques, including our own reported here, also corroborate the findings from isolated FCMs studied *ex vivo*, but these have so far relied almost entirely on immunohistochemistry. In the future, it would be desirable to confirm them with other methods such as transcriptomics of laser capture dissected plaque cells. However, this will not be an easy task, owing to the limited amounts and quality of extracted RNA, the difficulties of extracting FCMs and NFMs from the same microenvironments, and also of distinguishing macrophages from smooth muscle cells based on CD68 staining [6].

In addition, although our differential gene analysis implicated pro-fibrotic signalling pathways related to PDGF and TGFβ, their precise roles will require further verification. Macrophage-selective knockout of individual pro-fibrotic mediators and their receptors would shed additional light but they are clearly beyond the present scope of this study. The translational potential of our studies also needs further consideration. It will be interesting to investigate the roles of the pro-fibrotic genes we identified in mice, using bio-bank and genetic approaches in man. From a treatment perspective, selective oestrogen receptor modulators such as tamoxifen that are known to increase active TGFβ levels in patients appear to stabilize plaques and reduce acute coronary syndromes [75,76]. Based on our findings, further approaches, including targeting production of FOS and JUN transcription factors might represent alternative strategies.

### Implications for plaque rupture

Our novel data show conclusively that FCMs overexpress mRNAs for collagen and other matrix proteins that would tend to stabilize plaques. At first sight, this conclusion is paradoxical and needs to be reconciled with the more commonly-held view that FCMs overexpress matrix-degrading enzymes and therefore promote collagen degradation and plaque rupture. However, it is worth remembering that arterial fatty streaks and other xanthomas that contain few VSMCs or fibroblasts neither rupture nor cause thrombosis. Our conclusion is, therefore, that formation of FCMs is intrinsically pro-fibrotic and this may be necessary to stabilize early lesions. FCMs in more advanced plaques, for example at the vulnerable shoulder regions of plaques, are exposed to locally-acting inflammatory stimuli, which initiate other transcriptional programmes that tip the balance from collagen synthesis to degradation and therefore promote plaque rupture.

## Supporting Information

S1 File Experiments using RAW 264.7 and mouse BMDM, including comparisons of RNA (Table A) and protein expression (Table B). (DOCX)

S1 Table Genes differentially regulated between FCM and NFM (Ingenuity array, n = 4). (XLS)
