# Supplementary material for: Foam Cell Formation In Vivo Converts Macrophages to a Pro-Fibrotic Phenotype
Source: PLoS One. 2015 Jul 21;10(7):e0128163. doi: 10.1371/journal.pone.0128163 (PMC4510387; doi:10.1371/journal.pone.0128163)
Supplement: S1 File — (DOCX) [file pone.0128163.s001.docx]

**File S1: Experiments using RAW 264.7 and BMDM**

***Materials and methods***

*RAW 264.7 cell culture.* Cells from the mouse monocyte/macrophage cell line RAW 264.7 (ATCC, UK) were seeded onto coverslips at 0.8 x 10^4^ cells/well and grown for 24 h in DMEM (Life Technologies, UK) supplemented with antibiotics, glutamine and 10% foetal calf serum (FCS) (Life Technologies). The cells were then washed in serum free media (SFM) and exposed to 0, 1, 10 or 20 ng/ml ng/ml human TGFβ1 (R&D Systems, USA) (doses established in previous studies [1,2]), for 0.75 or 18 h before being washed, fixed in 3% paraformaldehyde, dried and stored at -20°C until use. The efficacy of human TGFβ1 on mouse cells has been previously documented (http://www.rndsystems.com/Products/7754-BH). RAW cells were also plated into 24-well plates at 0.5 x 10^6^ cells/well and grown in DMEM + 10% FCS for later RNA and protein isolation. The media in some plates were changed to SFM at 8 h (serum-starved RAW: ssRAW). Twenty-four h after plating or serum starving, the cultures were washed in SFM and the cells exposed to 0, 1, 10 or 20 ng/ml hTGFβ1 for 0.17, 0.75, 1.5, 3, 6 or 18 h (with or without the addition of 10 μM proteasome inhibitor MG132 (Sigma) 0.5 h prior to TGFβ1 treatment).

*Bone marrow monocyte isolation and differentiation to bone marrow-derived macrophages (BMDM).* Mouse femurs and tibias were excised from C57BL/6 mice (Charles River, UK) on a normal diet. The bone ends were cut and bone marrow was flushed out with sterile PBS. Pelleted white blood cells were counted and plated into 24-well plate at 0.5 x 10^6^ cells/well for RNA and 12-well plate at 2 x 10^6^ cells/well for protein and grown in RPMI 1640 media (Life Technologies) + 20% FCS in the presence of 20 ng/ml recombinant human macrophage colony stimulating factor (M-CSF, R&D Systems). Some cells were also grown on coverslips. The media was changed every 3-4 days. After 7 days, when the cells had differentiated into macrophages, M-CSF was removed and the cells exposed to 0, 1, 10 or 20 ng/ml hTGFβ (with or without MG132) for 0.75 or 18 h in SFM. Cells were mixed in SDS lysis buffer for protein analysis or stored in RLT buffer for later RNA isolation.

*Immunocytochemistry and (immuno)histochemistry.* See main text.

*Western blotting.* Protein from RAW or BMDM preparations was measured using the Micro BCA protein assay kit (Thermo Scientific, UK), and loaded onto 10% SDS-PAGE gels at equal amounts of protein/lane (10-20 μg/lane), with one lane reserved for a protein ladder (Precision Plus Protein Dual Color Standards, Bio-Rad, UK). Proteins were transferred to a PVDF membrane (Immobilon-P Transfer Membrane, Millipore, USA), blocked in milk and probed with the antibody of interest (Main text Table 1, biglycan-GAPDH). Appropriate horseradish peroxidase (HRP)-labelled secondary antibodies (Main text Table 1) were used for detection, and the bands visualised using Immobilon Western Chemiluminescent HRP substrate (Millipore). Blots were stripped with striping solution (Re-blot Plus, Millipore) or HRP activity blocked using azide before re-probing.

***Results***

*Effect of TGFβ1 on RNA expression in mouse macrophages.* We compared the RNA expression of TGFβ1-treated RAW (n=3-6) and BMDM (n=3-5) with foam cell macrophages (FCM) and non-foamy macrophages (NFM) produced in vivo from ApoE null mice fed a high-fat diet or C57Bl/6 (WT) mice fed a normal diet (these cells were not cultured or exposed to TGFβ1 in vitro) (n=5-7), and present these results in Table S1 (in File S1). Some responses from the cells produced in vivo were similar to the cells treated with TGFβ1 in vitro, suggesting that there may have been some TGFβ1 locally produced within the sponge in vivo. However, not all changes in expression in the cells produced in vivo were the same as the cultured cells, and, in addition, the BMDM and RAW did not always respond the same way to TGFβ1.

**Table A. RNA expression in macrophages produced in vivo compared with expression in RAW and BMDM treated with TGFβ1 in vitro**

| **Gene** | **FCM / NFM** | **BMDM ± hTGFβ1**  **(0-20 ng/ml)** | **RAW ± hTGFβ1**  **(0-20 ng/ml)** | **serum-starved RAW ± hTGFβ1 (0-20 ng/ml)** |
| --- | --- | --- | --- | --- |
| SMAD2 | NS ↓ in FCM | NS ↑ with TGFβ1 @ 18 h  NC dose response  NC with time | NS ↑ with TGFβ1 @ 18 h  NC with time |  |
| SMAD3 | NS ↓ in FCM | **Sig ↓@ 20 ng/ml TGFβ1**  NS ↓ dose response | NC with TGFβ1  NC with time |  |
| cFos | **Sig ↑ in FCM** | **Sig ↑ with** **TGFβ1**  NS dose response | **sig ↑ @ 18 h TGFβ1,** NS ↑ @ 0.75 h  NS ↓ with time | **Sig ↑ @ 0.75 h,** NS ↑ @ 18 h with TGFβ1  **Sig dose response (@ 0.75 and 18h)**  NS ↓ with time |
| FosB | **Sig ↑ in FCM** | not much present, NC | not much present, NC |  |
| Jun | **Sig ↑ in FCM** | **↓ dose response (sig @ 20 ng/ml)** | NC with TGFβ1  NC with time | NS ↑ with TGFβ1  NS ↑ dose response  **↑ with time (sig @ 0 and 1 ng/ml)** |
| JunB | **Sig ↑ in FCM** | NC with TGFβ1  NC dose response | NC with TGFβ1  NC with time |  |
| LXRα | **Sig ↑ in FCM** |  | not much present, NC |  |
| TGFβ1 | NS ↑ in FCM | not much present, NS ↑ with TGFβ1 | NC with TGFβ1  NC with time |  |
| hCTGF | **Sig ↑ in FCM** | not much present, NS ↑ with TGFβ1 | not much present |  |
| BGN | **Sig ↑ in FCM** | NS ↑ with TGFβ1  NS ↑ with time | not much present | not much present,  NC with TGFβ1  NS ↓ dose response @ 18 h  NC time. |
| DCN | **Sig ↑ in FCM** | NS ↑ with TGFb1  NS ↑ with time |  |  |
| Col1a1 | **Sig ↑ in FCM** |  | not much present, NC | not much present, NC |
| Col6a1 | **Sig ↑ in FCM** | not much present NS ↑with TGFβ1, 18 h |  | NS ↑ @ 0.75 h with TGFβ1  **Sig ↓ @ 18 with TGFβ1**  NC with time |
| Thbs1 | **Sig ↓ in FCM** | **Sig ↓ with TGFβ1 @ 18 h** (except 1 ng/ml) | not much present,  NS ↑with TGFβ1  NC with time | not much present,  NC with TGFb1  NC dose response  NC time |
| CTSC | **Sig ↑ in FCM** | NC with TGFβ1  NC dose response | **Sig ↑ with TGFβ1 @ 18h** (NS ↓ @ 0.75 h)  Sig ↓ with time |  |
| ALK2 | NS ↑ in FCM | NC with TGFβ1  NC dose response |  | NC with TGFβ1  NC dose response  NC with time |
| ALK5 | NS ↓ in FCM | **Sig ↑with TGFβ1 @ 18h**  NC dose response | **Sig ↑with TGFb1 @ 18h**  **↑ dose response @18h**  **sig ↑ with time** |  |
| SCARB1 | **Sig ↑ in FCM** | **Sig ↑with TGFβ1 @ 18h (20 ng/ml)** | NS ↑with TGFb1 18h  NC with time |  |
| CD36 (SCARB3) | NS ↑ in FCM | NC with TGFβ1 | NS ↑with TGFb1 @ 18 h  NC with time |  |

FCM = foam cell macrophage (from sponge granuloma), NFM = non-foamy macrophages (from sponge granuloma). NS = not significant; NC = no change; DS = dose response, grey = not done. Bold indicates significant difference compared with NFM or control.

*Effect of TGFβ1 on protein expression in mouse macrophages.* BMDM (n=5) and non-serum starved RAW cells (n=3) were examined for their response to TGFβ1 (Table S2 in File S1). Product degradation in some experiments was prevented using the proteasome inhibitor MG132. Interestingly, the use of the same antibody in western blotting and immunocytochemistry experiments on sister cultures of cells did not always lead to the same response being observed (eg CTGF, cFOS).

**Table B. Protein expression in RAW and BMDM treated with TGFβ1 in vitro**

| **Protein** | **Cell type** | **Effect of TGFb1** | | **Effect of time** | | | **ICC summary** | |
| --- | --- | --- | --- | --- | --- | --- | --- | --- |
|  |  | **WB** | **ICC** | **WB time course** | **WB** | **ICC** | **0.75 h** | **18 h** |
| BGN | RAW | NS |  | late | NS |  |  |  |
| CTGF | RAW | ↓ | ↑ | late | ↑ | ↑ | High and low dose: cytoplasmic staining. Associated with spindle | High and low dose: cytoplasmic staining |
| cFOS | RAW | ↕ | ↑ | early | ↑ | ↓ | High and low dose: nuclear and cytoplasmic staining | High dose: cytoplasmic staining. Low dose: no staining |
| cFOS | BMDM |  |  |  |  |  | Cytoplasmic staining only |  |
| pSMAD2 | RAW | ↑ | ↑ | early | ↓ |  | nuclear and cytoplasmic staining |  |
| pSMAD2 | BMDM | ↑ |  |  | ↓ |  |  |  |
| pSMAD3 | RAW |  |  |  |  |  | nuclear and cytoplasmic staining |  |
| pSMAD3 | BMDM | NS |  |  | NS |  |  |  |
| LXRα | RAW | ↓ |  | early | NS |  |  |  |

Band density was normalised to GAPDH. WB = western blotting, ICC = immunocytochemistry. NS = not significant, grey = not done.

**References**

1. Zuckerman SH, Panousis C, Evans G (2001) TGF-β reduced binding of high-density lipoproteins in murine macrophages and macrophage-derived foam cells. Atherosclerosis 155: 79-85.

2. Kalinina N, Agrotis A, Antropova Y, Ilyinskaya O, Smirnov V, Tararak E, Bobik A (2004) Smad expression in human atherosclerotic lesions. Evidence for impaired TGF-β/Smad signaling in smooth muscle cells of fibrofatty lesions. Arterioscler Thromb Vasc Biol 24: 1391-1396.
